# Suicide Risk and Protective Factors Among Medicaid-Enrolled Black Youth With a Mental Health Diagnosis

**DOI:** 10.1001/jamanetworkopen.2025.59657

**Published:** 2026-02-18

**Authors:** Cynthia A. Fontanella, Xueting Xia, Elyse N. Llamocca, Danielle L. Steelesmith, Guy N. Brock, Yunyu Xiao, Jeffrey A. Bridge, Andrea Young, John V. Campo, Donna Ruch

**Affiliations:** 1 Center for Suicide Prevention and Research at Nationwide Children’s Hospital, Columbus, Ohio; 2 Department of Psychiatry and Behavioral Health, The Ohio State University College of Medicine, Columbus; 3 Department of Bioinformatics, The Ohio State University College of Medicine, Columbus; 4 Weill Cornell Medical College, New York, New York; 5 Department of Pediatrics, The Ohio State University College of Medicine, Columbus; 6 Bloomberg School of Public Health, Johns Hopkins, Baltimore, Maryland; 7 Department of Psychiatry and Behavioral Science at Johns Hopkins University School of Medicine, Baltimore, Maryland

## Abstract

**Importance:**

Suicide rates among Black youth have risen sharply in recent years, surpassing those of other racial and ethnic groups. However, research examining factors contributing to suicide in this population remains limited.

**Objective:**

To examine individual-, family-, and contextual-level factors associated with suicide death among Black youth with a lifetime mental health diagnosis.

**Design, Setting, and Participants:**

This population-based case-control study examined US Medicaid data of 875 Black youth who died by suicide between January 1, 2010, and December 31, 2019. Each suicide case was matched with 10 living controls (ie, 8750 individuals) on age, sex, and state. All cases and controls were Medicaid-enrolled and had a documented lifetime mental health diagnosis. Data were analyzed from March to December 2025.

**Exposures:**

Individual (demographic, clinical characteristics, service history), family (history of abuse and neglect, family relational problems, and economic and housing problems), and contextual variables (social vulnerability, urban-rural status, crime rates, religious institution density) were abstracted from Medicaid claims data and supplemented with county-level data from publicly available sources.

**Main Outcomes and Measures:**

Suicide deaths, identified from National Death Index records. Hierarchical generalized estimating equations were fit to examine factors associated with suicide.

**Results:**

The total sample of 9625 individuals had a mean (SD) age of 18.9 (3.6) years and was primarily male (6950 [72.2%]). Factors associated with increased odds of suicide included foster care (aOR, 1.81 [95% CI, 1.34-2.44]) and disability status (aOR, 1.23 [95% CI, 1.01-1.49]) vs poverty enrollment in Medicaid, depression (aOR, 1.94 [95% CI, 1.59-2.38]) and schizophrenia or psychosis diagnoses (aOR, 3.52 [95% CI, 2.68-4.62]), prior deliberate self-harm (aOR, 11.01 [95% CI, 7.32-16.55]), prior acute mental health care (aOR, 2.20 [95% CI, 1.37-3.54]), brain injury (aOR, 4.41 [95% CI, 2.55-7.64]), violence exposure (aOR, 2.65 [95% CI, 1.51-4.65]), family relational problems (aOR, 2.27 [95% CI, 1.18-4.38]), or living in an urban community (aOR, 1.79 [95% CI, 1.40-2.29]) or in a moderate socially vulnerable county (aOR, 1.39 [95% CI, 1.13-1.73]) or high socially vulnerable county (aOR, 1.45 [95% CI, 1.17-1.80]). Factors associated with decreased odds included a diagnosis of anxiety (aOR, 0.70 [95% CI, 0.53-0.93]) and developmental disorders (aOR, 0.45 [95% CI, 0.29-0.69]) and living in a county with moderate (aOR, 0.78 [95% CI, 0.65-0.95]) or high (aOR, 0.65 [95% CI, 0.47-0.90]) rates of religious institutions. The highest population attributable risk (PAR) for suicide was prior deliberate self-harm (PAR, 56.4%; 95% CI, 53.5%-58.3%).

**Conclusions and Relevance:**

In this case-control study of Medicaid-enrolled Black youth with a lifetime mental health diagnosis, suicide risk was driven by a complex interplay of individual, family, and contextual factors. These results highlight the importance of culturally responsive, community-rooted, and equity-focused prevention strategies to reduce suicide risk in Black youth.

## Key Points

**Question:**

What factors are associated with suicide among Medicaid-enrolled Black youth with a lifetime mental health diagnosis?

**Findings:**

In this case-control study of 9625 youth, suicide risk was higher among Black youth with depression, psychosis, prior suicide attempts, psychiatric acute care, brain injury, family conflict, violence exposure, and placement in foster care or Medicaid eligibility due to disability status. Contextual-level risk was elevated in urban (vs rural) and moderate and high social vulnerability areas (vs low) and reduced in areas with moderate and high densities of religious institutions (vs low).

**Meaning:**

These findings suggest the need for testing effectiveness of multilevel suicide prevention strategies that address individual, family, and contextual factors for Black youth.

## Teaser

This case-control study examines individual-, family-, and contextual-level factors associated with suicide death among Black youth with a lifetime mental health diagnoses using US Medicaid data.

## Introduction

Suicide among Black children, adolescents, and young adults (collectively termed *youth*) is a public health crisis in the US. In 2023, suicide was the third leading cause of death for Black youth aged 10 to 24 years, exceeding deaths from several major medical diseases combined.^1^ Black children aged younger than 13 years die by suicide at nearly twice the rate of their White peers.^2^ The suicide rate among Black youth more than doubled between 2010 and 2023 (4.86 to 10.21 per 100 000), increasing faster than in any other racial or ethnic group.^3^ These trends spurred the Congressional Black Caucus’s 2019 report *Ring the Alarm: The Crisis of Black Youth Suicide in America*, calling for more research to understand and address suicide risk among Black youth.^4^ Yet research on factors associated with suicide among Black youth remains limited.^5^

The Social-Ecological Suicide Prevention Model (SESPM),^6^ suggests that suicide risk among Black youth arises from interacting individual-, family-, and contextual-level factors. General risk factors for youth suicide, such as older age, male sex, psychiatric disorders, prior suicidal behavior, bullying, impaired family functioning, sexual and gender minority status, and lethal means access, apply broadly to all youth.^7,8^ However, Black youth traditionally have more exposure to trauma, adverse childhood experiences, structural racism, discrimination, and disparities in access to mental health care.^9,10^ Additionally, Black youth are disproportionately affected by adverse social determinants of health (SDOH), including poverty, housing instability, and food insecurity, which worsen mental health and increase suicide risk.^11,12,13,14^ Although neighborhood disadvantage is linked to suicide risk in adults, fewer studies have examined its effects on Black youth.^15,16,17^ Many Black youth live in underresourced neighborhoods with high crime, violence, and environmental stressors that may heighten suicide risk.^18,19^ Structural barriers to care—clinician shortages, stigma, and culturally unresponsive services—can also delay or prevent treatment.^20^

Protective factors against suicide risk among Black youth remain understudied.^9^ Emerging evidence suggests that strong family support, positive ethnic identity, spiritual engagement, and community connectedness are key protective factors.^21^ Identifying and strengthening these factors is essential for developing effective, culturally responsive interventions.

This study examines multilevel risk and protective factors associated with suicide among Medicaid-enrolled Black youth with a lifetime mental health diagnosis. We focus on this high-risk population with intersecting vulnerabilities for several reasons. First, Medicaid is an especially important boundary setting covering more than 38 million children annually and serving populations with higher concentrations of mental illness and suicide risk.^22,23^ Black youth are also disproportionately represented in Medicaid and often face social and economic challenges that affect both health and health care navigation.^24^ Second, suicide risk in young people is substantially elevated across nearly all mental health disorders, with estimates indicating a 5- to 15-fold increase in risk.^25^ Finally, youth with mental health diagnoses interact more frequently with the health care system, creating intervention touchpoints for detection, risk assessment, and early intervention. Given these intersecting vulnerabilities, our focus on Black youth allows for deeper understanding of intragroup patterns and correlates of suicide in a high-risk population, rather than across-group disparities, to inform targeted, culturally responsive suicide prevention strategies.

## Methods

### Study Design and Sample

We conducted a population-based, matched case-control study of non-Hispanic Black Medicaid-enrolled participants aged 9 to 24 years in all 50 states and Washington, DC, between January 1, 2010, and December 31, 2019, with a lifetime mental health diagnosis (anxiety, posttraumatic stress disorder [PTSD], attention-deficit/hyperactivity disorder [ADHD], mood, disruptive behavior, psychotic, or substance use disorders; N = 3 592 457). These diagnoses were selected because they are the most common among youth. Data from Maryland (2016 to 2017) were excluded due to incomplete diagnostic information.^26^ We examined youth aged 9 to 24 years to examine both early-onset and later-emerging suicide risk. Race and ethnicity were self-reported. Cases were suicide decedents with 10 months or more of continuous Medicaid enrollment in the year before death.^27^ Each case was matched to 10 randomly selected controls on age, sex, and state of residence. Controls were alive on the index date (the case’s death date), had a lifetime mental health diagnosis, and met the same enrollment criteria as cases. Similar methods were used in prior suicide case-control studies.^28,29,30^ Comparison between included and excluded Medicaid enrollees is shown in eTable 1 in Supplement 1. All procedures were approved by The Ohio State University institutional review board and adhered to the Strengthening the Reporting of Observational Studies in Epidemiology (STROBE) reporting guideline.^31^ Informed consent was waived due to minimal risk and use of deidentified data collected for routine medical purposes.

### Data Sources

We used Medicaid Analytic eXtract (MAX) and Transformed Medicaid Statistical Information System Analytic File (TAF) from the Centers for Medicare & Medicaid Services, linked to the National Death Index (NDI). MAX and TAF included information on paid inpatient and outpatient services, enrollment, demographics, and place of residence. NDI, the reference standard for US mortality data, provided cause and date of death.^32^ Contextual-level information was linked from external sources using county-level Federal Information Processing Standards (FIPS) codes from MAX and TAF.

### Measures

#### Dependent Variable

The primary outcome was suicide death, identified using *International Statistical Classification of Diseases and Related Health Problems, Ninth *(*ICD-9*) and *Tenth Revision *(*ICD-10*) cause of death codes X60-X84, Y87.0, and *U03 as the underlying cause of death in the NDI, consistent with the US federal definition of suicide used by the US Centers for Disease Control and Prevention.^33^

#### Independent Variables

Our selection of individual, family, and contextual variables was guided by the SESPM, prior youth suicide research,^7,8^ and the availability of relevant measures in Medicaid claims and external data sources. As adverse SDOH are strongly linked to suicide risk, we included many of the *ICD-9*/*ICD-10* codes associated with these determinants as potential risk factors.^34,35,36,37^

#### Individual-Level Factors

Individual-level factors included Medicaid eligibility reason (disability, foster care, poverty, other); mental health and general medical conditions; prior deliberate self-harm (DSH); individual level SDOH variables; and acute or outpatient mental health care within 1 month before the index date. Mental health conditions included: ADHD, PTSD, anxiety, developmental disorders, bipolar, depressive, disruptive behavior-disorders, schizophrenia and related psychosis, substance use disorders, and other residual mental health disorders (eTable 2 in Supplement 1). General medical conditions included: asthma, epilepsy or seizure disorders, sleep disorders, and traumatic brain injury or concussion (TBI) (eTable 3 in Supplement 1); all have known associations with suicide.^30,38,39,40^ Mental health and medical conditions were defined as 2 or more claims with relevant *ICD-9*/*ICD-10* codes in the year before the index date. DSH (eTable 4 in Supplement 1), including both nonsuicidal self-injury and suicide attempts, and SDOH were identified using 1 or more claim in the year preceding the index date. SDOH variables included educational or occupational problems (eg, school difficulties, unemployment, and adverse work conditions) and exposure to violence (eg, assault or injury due to legal intervention) (eTable 5 in Supplement 1).^41,42,43,44,45^

Place of service codes were used to identify acute mental health care in the month before the index date, including inpatient psychiatric hospitalizations and emergency department visits with a primary mental health diagnosis. Outpatient mental health care was defined as any encounter with a primary mental health diagnosis or mental health procedure code (eg, diagnostic interview, medication management, psychotherapy, case management, crisis intervention) in an office, home, hospital, or similar setting.

#### Family-Level Factors

Family-level factors included 3 SDOH variables: child abuse or neglect history, family relational problems, and economic or housing problems (eTable 5 in Supplement 1). These were defined as 1 or more claim with a relevant *ICD-9*/*ICD-10* diagnosis code in the year before the index date.^41,42,43,44,45^

#### Contextual-Level Factors

Using Rural-Urban Continuum Codes (RUCC), county of residence was classified as urban (categories 1 to 3) or rural (categories 4 to 9).^46^ County-level capacity to respond to external stressors (eg, natural disasters) was measured using the Social Vulnerability Index (SVI).^47,48^ The SVI ranks US counties using a composite measure of social vulnerability based on 16 factors grouped into 4 domains: socioeconomic status, housing type and transportation, household characteristics, and racial and ethnic minority status. We categorized SVI scores into terciles based on national percentile ranking, with lower percentiles representing lower vulnerability: low (0 to <33.3), moderate (33.3 to <66.7), and high (≥66.7). We also categorized the number of religious organizations^49^ and county crime rates^50^ per 100 000 residents into terciles based on national rankings. Additional details are provided in eTable 6 in Supplement 1.

### Statistical Analysis

We summarized the sample using descriptive statistics. Associations between risk and protective factors and suicide were estimated using unadjusted and adjusted logistic regression models with odds ratios (ORs) estimated by generalized estimating equations while accounting for matching and county-level clustering by taking the intersection between matched sets and county. Nested adjusted models were fit in 3 hierarchical steps based on the SESPM^6^: (1) individual-level factors, (2) family-level factors, and (3) contextual-level factors. Variance inflation factors assessed multicollinearity. Multiple imputation (10 datasets) under multivariate model with discriminant function addressed missing contextual data. eTable 7 in Supplement 1 provides a summary of missingness of contextual variables. Model fit was evaluated using quasi information criterion (QIC).^51^ Population attributable risks (PARs) were calculated in R version 4.4.2 (R Project for Statistical Computing) for all significant variables in the final model that were possibly modifiable through intervention.^52^ PAR estimates the proportion of cases attributable to a specific exposure.^53^ All other analyses were conducted in SAS version 9.4 (SAS Institute) with significance set at 2-sided *P* < .05. Data were analyzed from March to December 2025.

## Results

The final sample included 9625 individuals: 875 suicide decedents (cases) and 8750 matched controls. Mean (SD) age at index date was 18.9 (3.6) years, and 6950 (72.2%) were male. The mean (SD) months of enrollment for all individuals was 11.9 (0.4) months, with no significant difference between cases (11.9 [0.5] months) and controls (11.9 [0.4] months). The most common suicide method was hanging or suffocation (381 [43.5%]), followed by firearms (278 [31.8%]) and poisoning (72 [8.2%]). Significant group differences were observed between cases and controls across individual, family, and contextual factors (Table 1 and Table 2).

**Table 1.** Individual and Family-Level Characteristics of Medicaid-Enrolled Black Youth and Young Adult Suicide Cases and Controls

Characteristic	Participant, No. (%)			OR (95% CI)
	Total (n = 9625)	Suicide cases (n = 875)	Controls (n = 8750)	
Individual characteristics				
Medicaid eligibility				
Poverty	5478 (56.9)	442 (50.5)	5036 (57.5)	1.00 [Reference]
Disability	2204 (22.9)	242 (27.7)	1962 (22.4)	1.41 (1.19-1.65)
Foster	487 (5.1)	68 (7.8)	419 (4.8)	1.85 (1.41-2.43)
Other^a^	1456 (15.1)	123 (14.1)	1333 (15.2)	1.05 (0.86-1.29)
Mental health diagnoses^b^				
ADHD	1488 (15.5)	119 (13.6)	1369 (15.7)	0.85 (0.69-1.04)
Autism spectrum and developmental disorders	569 (5.9)	26 (3.0)	543 (6.2)	0.46 (0.31-0.69)
Disruptive behavior disorders	648 (6.7)	83 (9.5)	565 (6.5)	1.52 (1.19-1.93)
Depressive disorders	1246 (12.9)	223 (25.5)	1023 (11.7)	2.58 (2.19-3.04)
Bipolar disorders	829 (8.6)	117 (13.4)	712 (8.1)	1.74 (1.41-2.15)
Anxiety disorders	864 (9.0)	89 (10.2)	775 (8.9)	1.17 (0.93-1.47)
PTSD	293 (3.0)	47 (5.4)	246 (2.8)	1.96 (1.43-2.69)
Schizophrenia and related psychosis	451 (4.7)	139 (15.9)	312 (3.6)	5.11 (4.13-6.32)
Substance use disorders	1012 (10.5)	164 (18.7)	848 (9.7)	2.15 (1.79-2.58)
Other mental health disorders^c^	1336 (13.9)	138 (15.8)	1198 (13.7)	1.18 (0.97-1.43)
General medical conditions^b^				
Asthma	440 (4.6)	47 (5.4)	393 (4.5)	1.21 (0.88-1.65)
Epilepsy or seizure	173 (1.8)	23 (2.6)	150 (1.7)	1.55 (0.99-2.42)
Sleep disorder	136 (1.4)	17 (1.9)	119 (1.4)	1.44 (0.86-2.40)
TBI or concussion	71 (0.7)	24 (2.7)	47 (0.5)	5.22 (3.17-8.60)
Prior deliberate self-harm^b^	149 (1.6)	89 (10.2)	60 (0.7)	16.40 (11.77-22.85)
Recent acute mental health care^c^	137 (1.4)	42 (4.8)	95 (1.1)	4.59 (3.17-6.66)
Recent outpatient mental health care^c^	1782 (18.5)	182 (20.8)	1600 (18.3)	1.17 (0.99-1.39)
Educational or occupational problem	58 (0.6)	11 (1.3)	47 (0.5)	2.36 (1.22-4.56)
Exposure to violence	81 (0.8)	21 (2.4)	60 (0.7)	3.56 (2.15-5.90)
Family characteristics				
History of child abuse or neglect	78 (0.8)	14 (1.6)	64 (0.7)	2.21 (1.24-3.92)
Family relational problem	71 (0.7)	18 (2.2)	53 (0.5)	3.45 (2.01-5.91)
Economic and housing problem	59 (0.6)	19 (2.2)	40 (0.5)	4.83 (2.79-8.39)

Abbreviations: ADHD, attention-deficit/hyperactivity disorder; OR, odds ratio; PTSD, posttraumatic stress disorder; TBI, traumatic brain injury.

^a^ Other includes unknown eligibility and eligibility groups, such as those covered for specific conditions and qualified noncitizens.

^b^ Based on diagnoses within Medicaid claims data for the year prior to index date.

^c^ Other mental health diagnoses include all *International Statistical Classification of Diseases and Related Health Problems, Ninth* and *Tenth Revision* codes 290-319 and F00-F99 not included in other categories.

**Table 2.** Contextual-Level Characteristics of Medicaid-Enrolled Black Youth and Young Adult Suicide Cases and Controls

Contextual characteristics	Participant, No. (%)			OR (95% CI)^a^
	Total (n = 8698)^b^	Suicide cases (n = 807)	Controls (n = 7891)	
RUCC				
Urban	7085 (81.5)	729 (90.3)	6356 (80.6)	2.24 (1.78-2.82)
Rural	1613 (18.5)	78 (9.7)	1535 (19.4)	1 [Reference]
Social vulnerability index^c^				
Low	1603 (18.4)	109 (13.5)	1494 (18.9)	1 [Reference]
Moderate	3658 (42.1)	360 (44.6)	3298 (41.8)	1.46 (1.19-1.79)
High	3437 (39.5)	338 (41.9)	3099 (39.3)	1.43 (1.16-1.75)
Religious establishments^c^				
Low	5785 (66.5)	613 (76.0)	5172 (65.5)	1 [Reference]
Moderate	2090 (24.0)	150 (18.6)	1940 (24.6)	0.68 (0.57-0.81)
High	823 (9.5)	44 (5.4)	779 (9.9)	0.45 (0.33-0.61)
Crime rates^c^				
Low	2293 (26.4)	193 (23.9)	2100 (26.6)	1 [Reference]
Moderate	3187 (36.6)	287 (35.6)	2900 (36.8)	1.09 (0.91-1.29)
High	3218 (37.0)	327 (40.5)	2891 (36.6)	1.22 (1.02-1.45)

Abbreviation: OR, odds ratio; RUCC, rural-urban continuum codes.

^a^ ORs computed based on model results from 10 imputations.

^b^ Table only includes individuals with complete data for all contextual-level variables due to missing categories with small cell sizes.

^c^ Cutoffs were made based on the 33.3 and 67.7 percentiles yearly.

### Individual-Level Factors

Suicide decedents had higher odds of Medicaid enrollment through disability (OR, 1.41 [95% CI, 1.19-1.65]) or foster care (OR, 1.85 [95% CI, 1.41-2.43]) compared to those enrolled through poverty. Decedents had higher odds of DSH (OR, 16.40 [95% CI, 11.77-22.85]), recent acute mental health care (OR, 4.59 [95% CI, 3.17-6.66]), TBI or concussion (OR, 5.22 [95% CI, 3.17-8.60]), educational or occupational problems (OR, 2.36 [95% CI, 1.22-4.56]), violence exposure (OR, 3.56 [95% CI, 2.15-5.90]), and the following disorders: bipolar (OR, 1.74 [95% CI, 1.41-2.15]), depressive (OR, 2.58 [95% CI, 2.19-3.04]), disruptive behavior (OR, 1.52 [95% CI, 1.19-1.93]), schizophrenia and related psychosis (OR, 5.11 [95% CI, 4.13-6.32]), substance use (OR, 2.15 [95% CI, 1.79-2.58]), and PTSD (OR, 1.96 [95% CI, 1.43-2.69]) compared with controls. Conversely, decedents had lower odds of developmental disorders than controls.

### Family-Level Factors

Compared with controls, suicide decedents had higher odds of child abuse or neglect history (OR, 2.21 [95% CI, 1.24-3.92]). Additionally, they had higher odds of family relational problems (OR, 3.45 [95% CI, 2.01-5.91]) and economic or housing problems (OR, 4.83 [95% CI, 2.79-8.39]).

### Contextual-Level Factors

Suicide decedents had higher odds of living in urban areas (OR, 2.24 [95% CI, 1.78-2.82]) compared with rural areas, a county with moderate (OR, 1.46 [95% CI, 1.19-1.79]) or high (OR, 1.43 [95% CI, 1.16-1.75]) social vulnerability compared with low social vulnerability, and a high crime rate (OR, 1.22 [95% CI, 1.02-1.45]) compared with a low crime rate. Decedents had lower odds of living in a county with moderate (OR, 0.68 [95% CI, 0.57-0.81]) or high (OR, 0.45 [95% CI, 0.33-0.61]) rates of religious establishments compared with low rates.

### Factors Associated With Suicide Among Black Youth

Table 3 presents results from adjusted models, with each step adding a new level of variables. Model 1 included individual-level factors. Youth enrolled in Medicaid via disability (adjusted odds ratio [aOR], 1.22 [95% CI, 1.01-1.48]) and foster care (aOR, 1.85 [95% CI, 1.37-2.48]) had higher odds of suicide than those enrolled based on poverty. Diagnoses of disruptive behavior (aOR, 1.35 [95% CI, 1.01-1.81]), depressive (aOR, 2.00 [95% CI, 1.64-2.44]), schizophrenia or psychosis (aOR, 3.78 [95% CI, 2.89-4.95]), substance use disorders (aOR, 1.25 [95% CI, 1.01-1.56]), and TBI (aOR, 4.38 [95% CI, 2.54-7.55]) were associated with increased odds of suicide, while developmental disorders (aOR, 0.46 [95% CI, 0.30-0.72]) and anxiety (aOR, 0.69 [95% CI, 0.52-0.91]) had lower odds of suicide. Prior DSH (aOR, 11.27 [95% CI, 7.57-16.78]) and recent acute mental health care (aOR, 2.12 [95% CI, 1.35-3.35]) were strong risk factors. Recent outpatient mental health care was associated with lower odds (aOR, 0.80 [95% CI, 0.65-0.99]), whereas violence exposure was associated with higher odds (aOR, 2.71 [95% CI, 1.55-4.76]). Model 1 had QIC of 5368.63.

**Table 3.** Estimated ORs of Factors Associated With Suicide Death Among Medicaid-Enrolled Black Youth and Young Adults

Outcomes	Adjusted OR (95% CI)		
	Model 1	Model 2	Model 3^a^
Individual characteristics			
Medicaid eligibility			
Poverty	1.00 [Reference]	1.00 [Reference]	1.00 [Reference]
Disability	1.22 (1.01-1.48)	1.24 (1.02-1.50)	1.23 (1.01-1.49)
Foster	1.85 (1.37-2.48)	1.86 (1.38-2.50)	1.81 (1.34-2.44)
Other^b^	1.06 (0.86-1.31)	1.07 (0.86-1.32)	1.06 (0.86-1.31)
Mental health diagnoses^b^			
ADHD	0.89 (0.71-1.11)	0.89 (0.71-1.11)	0.93 (0.75-1.16)
Autism spectrum and developmental disorders	0.46 (0.30-0.72)	0.45 (0.29-0.70)	0.45 (0.29-0.69)
Disruptive behavior disorders	1.35 (1.01-1.81)	1.33 (0.99-1.78)	1.34 (1.00-1.80)
Depressive disorders	2.00 (1.64-2.44)	1.96 (1.60-2.39)	1.94 (1.59-2.38)
Bipolar disorders	0.90 (0.68-1.19)	0.88 (0.67-1.17)	0.89 (0.67-1.19)
Anxiety disorders	0.69 (0.52-0.91)	0.69 (0.52-0.92)	0.70 (0.53-0.93)
PTSD	1.00 (0.66-1.51)	1.00 (0.66-1.52)	1.01 (0.66-1.54)
Schizophrenia and related psychosis	3.78 (2.89-4.95)	3.72 (2.84-4.86)	3.52 (2.68-4.62)
Substance use disorders	1.25 (1.01-1.56)	1.24 (0.99-1.54)	1.23 (0.99-1.54)
Other mental health disorders^c^	0.85 (0.67-1.08)	0.84 (0.66-1.07)	0.85 (0.67-1.09)
General medical conditions^d^			
Asthma	0.82 (0.57-1.18)	0.81 (0.57-1.17)	0.77 (0.54-1.11)
Epilepsy or seizure	1.25 (0.78-2.02)	1.23 (0.76-2.00)	1.19 (0.73-1.94)
Sleep disorder	1.03 (0.56-1.89)	1.01 (0.55-1.86)	1.03 (0.54-1.95)
TBI or concussion	4.38 (2.54-7.55)	4.38 (2.53-7.57)	4.41 (2.55-7.64)
Prior deliberate self-harm^d^	11.27 (7.57-16.78)	11.25 (7.52-16.83)	11.01 (7.32-16.55)
Recent acute mental health care^c^	2.12 (1.35-3.35)	2.09 (1.32-3.30)	2.20 (1.37-3.54)
Recent outpatient mental health care^c^	0.80 (0.65-0.99)	0.81 (0.66-1.00)	0.83 (0.67-1.02)
Educational or occupational problem	1.49 (0.65-3.42)	1.21 (0.49-2.97)	1.06 (0.43-2.62)
Exposure to violence	2.71 (1.55-4.76)	2.72 (1.55-4.77)	2.65 (1.51-4.65)
Family characteristics			
History of child abuse or neglect	NA	0.87 (0.38-1.97)	0.86 (0.38-1.96)
Family relational problem	NA	2.18 (1.11-4.28)	2.27 (1.18-4.38)
Economic and housing problem	NA	1.90 (0.91-3.99)	1.86 (0.88-3.89)
Contextual characteristics			
RUCC			
Urban	NA	NA	1.79 (1.40-2.29)
Rural	NA	NA	1.00 [Reference]
Social vulnerability index^e^			
Low	NA	NA	1.00 (1.00)
Moderate	NA	NA	1.39 (1.13-1.73)
High	NA	NA	1.45 (1.17-1.80)
Religious establishments^e^			
Low	NA	NA	1.00 (1.00)
Moderate	NA	NA	0.78 (0.65-0.95)
High	NA	NA	0.65 (0.47-0.90)
Crime rates^e^			
Low	NA	NA	1.00 (1.00)
Moderate	NA	NA	0.98 (0.81-1.19)
High	NA	NA	1.12 (0.93-1.36)
Model fit criteria: QIC	5368.63	5367.16	5311.05^f^

Abbreviations: ADHD, attention-deficit/hyperactivity disorder; NA, not available; OR, odds ratio; PTSD, posttraumatic stress disorder; QIC, quasi information criterion; RUCC, rural urban continuum codes; TBI, traumatic brain injury.

^a^ ORs computed based on model results from 10 imputations.

^b^ Other includes eligibility groups such as those covered for specific conditions and qualified noncitizens.

^c^ Other mental health diagnoses include all *International Statistical Classification of Diseases and Related Health Problems, Ninth* and *Tenth Revision* codes 290-319 and F00-F99 not included in other categories.

^d^ Based on diagnoses within Medicaid claims data for the year prior to index date.

^e^ Cutoffs were made based on the 33.3 and 67.7 percentiles yearly.

^f^ Average QIC based on QIC from 10 imputations, range: 5309.71-5312.44.

Model 2 added family-level variables. Only family relational problems were significantly associated with increased odds of suicide (aOR, 2.18 [95% CI, 1.11-4.28]). Significant associations from previous models persisted for most factors except for disruptive behavior and substance use disorders, and recent outpatient mental health care becoming nonsignificant. Model fit improved (QIC = 5367.16).

Model 3 added contextual-level factors. Estimates for individual- and family-level variables remained stable. Youth living in urban areas (aOR, 1.79 [95% CI, 1.40-2.29]) had higher odds of suicide compared with those living in rural areas. Residence in counties with moderate (aOR, 1.39 [95% CI, 1.13-1.73]) or high (aOR, 1.45 [95% CI, 1.17-1.80]) social vulnerability had higher odds of suicide than low social vulnerability counties. Residence in counties with moderate (aOR, 0.78 [95% CI, 0.65-0.95]) or high (aOR, 0.65 [95% CI, 0.47-0.90]) religious organization density had lower odds of suicide than counties with low religious organization density. Model 3 had the best overall fit, with a mean QIC of 5311.05 across imputed datasets.

### Population Attributable Risk

The Figure shows population attributable risks (PARs) for modifiable factors significantly associated with suicide in Model 3. Prior DSH had the highest PAR, with 56.4% (95% CI, 53.5%-58.3%) of suicides attributable to DSH history, followed by TBI (PAR, 25.4% [95% CI, 20.0%-28.5%]), schizophrenia or psychosis (PAR, 23.4% [95% CI, 20.5%-25.6%]), recent acute mental health care (PAR, 17.6% [95% CI, 8.8%-23.2%]), violence exposure (PAR, 16.9% [95% CI, 9.1%-21.3%]), and family relational problems (PAR, 13.3% [95% CI, 3.6%-18.4%]), respectively. Other significant factors each had PARs of less than 10%.

**Figure.**
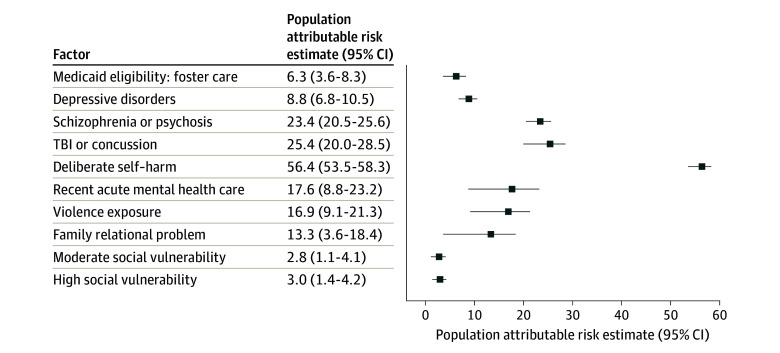
Forest Plot of Population Attributable Risks for Modifiable Individual-, Family-, and Contextual-Level Factors Associated With Suicide Among Black Youth TBI indicates traumatic brain injury.

## Discussion

To our knowledge, this is the first large-scale national study to examine multilevel risk and protective factors for suicide among Medicaid-enrolled Black youth with a lifetime mental health diagnosis. Our findings indicate that suicide risk in this population is shaped by a constellation of individual factors, including demographic characteristics, clinical profiles, and service use history, as well as family and broader contextual factors. These results underscore the complex interplay of clinical, social, and structural factors contributing to suicide risk in Black youth and highlight the need for multidimensional, culturally informed prevention strategies.

A history of DSH was the most potent risk factor for suicide, with over 11-fold increased odds and a PAR of 56%, suggesting that more than half of suicide deaths might theoretically be prevented if self-harm were eliminated. This finding is consistent with extensive prior research demonstrating that DSH history is one of the strongest suicide risk factors across youth populations.^7,54^ Timely assessment and sustained follow-up after DSH is particularly important in this high-risk population, regardless of perceived lethality.^55^

Consistent with prior research,^7^ psychiatric diagnoses were among the strongest risk factors associated with suicide. Black youth diagnosed with schizophrenia or psychosis had more than 3 times the odds of dying by suicide, with a PAR of 23%; those with depression had nearly twice the odds, with a PAR of 9%. Schizophrenia and psychosis are well-known suicide risk factors, with 5% to 13% of affected individuals dying by suicide.^56,57^ The link between depression and suicide is also well established; 60% to 70% of youth who die by suicide experience depressive symptoms, and up to 90% of adolescents who attempt suicide have a depression diagnosis.^58,59^ Routine suicide risk screening, access to effective high-intensity treatments,^60,61,62^ along with safety planning, family involvement, and lethal means reduction, remain essential components of care.^63,64^

Our finding of anxiety disorders being associated with lower odds of suicide death diverges from a recent meta-analysis that reported no significant association between anxiety disorders and suicide in a mixed sample of adolescents and adults.^65^ One possibility, given all participants in this study had at least 1 lifetime mental health or substance abuse condition, is that a heightened fear of death or increased risk aversion—features common in some anxiety disorders—may reduce the likelihood of acting on suicidal thoughts compared with peers with other psychiatric conditions, such as mood or externalizing disorders, that may involve greater hopelessness or impulsivity.^65,66^

The finding that Black youth with developmental disorders had lower suicide risk is unexpected and differs from prior research.^67,68,69,70^ Suicide risk in this population is likely heterogeneous, given wide variation in cognitive functioning, communication abilities, adaptive skills, and comorbid psychiatric conditions, although some individuals may have diminished capacity to engage in high-lethality suicidal behaviors. Further research is needed to clarify the association between developmental disorders and suicide risk.

In contrast to the lower risk observed for developmental disorders, TBI was a substantial risk factor for suicide in Black youth, with a 4-fold increased odds of suicide, and a PAR of 25%. This finding aligned with previous research demonstrating increased suicide risk following head trauma^71^ through its effects on emotional regulation, impulsivity, and co-occurring psychiatric symptoms.^72^ Because TBI is relatively common, especially among student athletes, education about this risk is essential. Clinicians treating youth with TBI should assess suicide risk and connect patients to appropriate mental health care when indicated.

Suicide risk was also elevated following recent acute psychiatric care, with a 2.2-fold increase in odds and a PAR of 18%. Although these encounters often entail short-term stabilization, the transition back to the community is a well-established period of elevated suicide risk.^73,74^ Prior research suggested that Black patients were less likely to receive timely follow-up, which may reflect cultural beliefs, stigma, and systemic inequities, including clinician bias and lack of access to culturally responsive care.^14^ Improved continuity of care (eg, postdischarge outreach, care navigation) may prove helpful and warrants additional study.

Consistent with existing research, including a meta-analysis showing^75^ exposure to violence was associated with a 10-fold increase for suicide death among youth, Black youth exposed to violence in our study had nearly 3-fold higher odds of suicide death, with a PAR of 17%. Prior studies also indicated that Black youth experience violence at much higher rates than their White peers.^76^ Developing best practices to identify Black youth exposed to violence can improve suicide risk assessment and intervention. One promising school-based suicidal ideation preventive intervention is the Adapted Coping with Stress Course (A-CWS),^77^ designed to strengthen Black adolescents’ coping skills with socioecological stressors (eg, community violence exposure). A-CWS is especially effective in youth with higher baseline suicidal ideation.

Medicaid eligibility due to disability status and foster care was associated with higher odds of suicide compared with eligibility from poverty. Disabled Black youth were more likely to die by suicide, which may reflect the cumulative impact of co-occurring mental health conditions, functional limitations, and barriers to accessing timely and appropriate behavioral health care. Black youth are overrepresented in the child welfare system^78,79^ and risk is likely compounded by trauma, disrupted attachments, and placement instability. These findings highlight the need for trauma-informed, youth-centered suicide prevention strategies embedded within child welfare systems.

Family relational problems were associated with suicide after adjusting for individual-level factors. Black youth with such difficulties had 2-fold higher odds of suicide, with a PAR of 13%. Existing evidence shows that strengthening family relations and connectedness^80,81^ are important interventions to reduce youth suicidal behavior. Empirically supported family-based approaches, such as Attachment-Based Family Therapy^82^ and the Family Intervention for Suicide Prevention/Safety-Acute (A),^83,84^ offer promising solutions to improve family discord and lower suicide risk.

Several contextual-level variables remained significantly associated with suicide even after controlling for individual- and family-level factors. Black youth living in urban areas and counties with higher social vulnerability were more likely to die by suicide. Social vulnerability reflects broader structural disadvantages that can increase stress and reduce access to supportive resources.^85,86^ These findings underscore the importance of addressing broader environments in which Black youth live and prioritizing community infrastructure investments, increased availability of services, and policies that reduce resource distribution inequities.

Conversely, Black youth living in areas with a higher density of religious organizations had a 30% lower risk of suicide. The presence of religious organizations may be a proxy for stronger community cohesion, greater availability of supportive social network, and increased opportunities to engage in faith-based or community-driven activities that promote resilience and belonging. This may be especially relevant in Black communities, where faith-based environments may offer emotional support, moral guidance, and crisis intervention, particularly in communities with limited formal mental health services.^87,88,89^ Leveraging faith-based organization partnerships and existing community strengths to promote protective social environments for at-risk youth may help prevent suicide. Future research must clarify the mechanisms by which faith-based and community organizations confer protection and determine how to effectively integrate these strengths into suicide prevention efforts for Black youth.

### Limitations

This study has several limitations. First, individual and family SDOH variables are commonly unreported within Medicaid claims data, and when documented, are often inconsistently recorded in clinical settings.^90^ Second, contextual factors, measured at the county level, may not accurately capture neighborhood-level variation or the lived experiences of individuals. Third, we were unable to assess important psychosocial factors—such as perceived discrimination, racial identity, social support, and stigma—that are not captured in claims but are highly relevant to suicide risk among Black youth. Fourth, diagnostic classification of psychiatric diagnoses is based on clinical judgment, not standardized diagnostic procedures. Fifth, claims records do not distinguish self-harm injuries with suicidal vs nonsuicidal intent and do not capture self-harm that does not result in medical care. Additionally, although concerns exist regarding validity^91^ and completeness^92^ of codes for identifying deliberate self-harm, prior research has demonstrated high concordance between self-harm E-codes and medical record documentation of intentional injury with suicidal intent.^93^ Sixth, suicide deaths among Black youth are often misclassified or undercounted, which may lead to underestimation of true suicide rates.^94,95,96^ Finally, findings may not be generalizable to Black youth with private insurance, those who are uninsured, or those without a lifetime mental health diagnosis.

## Conclusions

In this case-control study of Medicaid-enrolled Black youth with a lifetime mental health diagnosis, we identified individual, family, and contextual factors independently associated with suicide risk. Self-harm was the strongest factor, followed by schizophrenia or psychosis, depression, TBI, exposure to violence, foster care and disability status, and recent acute psychiatric care. Family relational problems and structural disadvantage indicators elevated risk, while a greater density of religious organizations showed protective associations. These findings highlight the need for culturally informed, equity-centered suicide prevention strategies that operate across levels of influence and emphasize early identification of at-risk youth.

## Notes

Supplement 1. **eTable 1. **Demographic Comparison of Selected Cohort and Removed Cohort Due to Enrollment **eTable 2. **Mental Health Variables and *International Statistical Classification of Diseases and Related Health Problems* (*ICD*) Codes **eTable 3. **Physical Health Variables and *International Statistical Classification of Diseases and Related Health Problems* (*ICD*) Codes **eTable 4. **Deliberate Self-Harm* International Statistical Classification of Diseases and Related Health Problems* (*ICD*) Codes **eTable 5. **Other Individual and Family Variables* International Statistical Classification of Diseases and Related Health Problems* (*ICD*) Codes **eTable 6. **Contextual Level Variable Sources and Yearly Availability **eTable 7. **Missingness of Contextual Level Variables

Supplement 2.
Data Sharing Statement
