# Supplementary material for: Suicide Risk and Protective Factors Among Medicaid-Enrolled Black Youth With a Mental Health Diagnosis
Source: JAMA Netw Open. 2026 Feb 18;9(2):e2559657. doi: 10.1001/jamanetworkopen.2025.59657 (PMC12917685; doi:10.1001/jamanetworkopen.2025.59657)
Supplement: Supplement 1. — eTable 1. Demographic Comparison of Selected Cohort and Removed Cohort Due to Enrollment eTable 2. Mental Health Variables and International Statistical Classification of Diseases and Related Health Problems (ICD) Codes eTable 3. Physical Health Variables and International Statistical Classification of Diseases and Related Health Problems (ICD) Codes eTable 4. Deliberate Self-Harm International Statistical Classification of Diseases and Related Health Problems (ICD) Codes eTable 5. Other Individual and Family Variables International Statistical Classification of Diseases and Related Health Problems (ICD) Codes eTable 6. Contextual Level Variable Sources and Yearly Availability eTable 7. Missingness of Contextual Level Variables [file jamanetwopen-e2559657-s001.pdf]

Supplemental Online Content

Fontanella CA, Xia X, Llamocca EL, et al. Risk and protective factors associated with suicide among Medicaid-enrolled black youth with a mental health diagnosis. *JAMA Network Open*. 2026. 9(2):e2558954. doi:10.1001/jamanetworkopen.2025.58954

eTable 1. Demographic Comparison of Selected Cohort and Removed Cohort Due to Enrollment

eTable 2. Mental Health Variables and *International Statistical Classification of Diseases and Related Health Problems* (*ICD*) Codes

eTable 3. Physical Health Variables and *International Statistical Classification of Diseases and Related Health Problems* (*ICD*) Codes

eTable 4. Deliberate Self-Harm *International Statistical Classification of Diseases and Related Health Problems* (*ICD*) Codes

eTable 5. Other Individual and Family Variables *International Statistical Classification of Diseases and Related Health Problems* (*ICD*) Codes

eTable 6. Contextual Level Variable Sources and Yearly Availability

eTable 7. Missingness of Contextual Level Variables

This supplemental material has been provided by the authors to give readers additional information about their work.

eTable 1. Demographic Comparison of Selected Cohort and Removed Cohort Due to Enrollment

Variable Name	Removed Cohort (N=41723)	Selected Cohort (N=9625)
	n (%)	n (%)
Age (SD)	19.6 (3.4)	18.9 (3.6)
Sex: male	29672 (71.1)	6949 (72.2)
Eligibility		
Poverty	36118 (88.6)	5478 (56.9)
Disability	2740 (6.7)	2204 (22.9)
Foster	425 (1.0)	487 (5.1)
Other	1497 (3.7)	1456 (15.1)

© 2026 Fontanella CA et al. *JAMA Network Open*.

eTable 2. Mental Health Variables and ICD Codes.

Diagnosis	ICD-9	ICD-10
Attention-deficit/hyperactivity disorder (ADHD)	314	F90
Autism spectrum and developmental disorders	299.0	F84.0, F84.5
	299, 315	F80-F89 except F84.0, F84.5
Disruptive behavior disorders	312, 313.81	F91
Depressive disorders	296.2, 296.3, 296.82, 300.4, 311	F32, F33, F34.1
Bipolar disorders	296.0, 296.1, 296.4-296.7, 296.80, 296.81, 296.89, 301.13	F30, F31, F34.0, except F30.3, F30.4, F31.7
	296.9	F34, F39, except F34.1
Anxiety	300.0-300.3, 309.21	F40-F42, F44
Post-traumatic stress disorder (PTSD)	309.81	F43.1
Schizophrenia and related pshchosis	295, 297, 298	F20-F29
Substance Use Disorder	291, 292, 303, 304, 305	F10-F19
Other mental health disorders	290-319, except the ones mentioned above	F00-F99, except the ones mentioned above

© 2026 Fontanella CA et al. *JAMA Network Open*.

eTable 3. Physical Health Variables and ICD Codes.

Diagnosis	ICD-9	ICD-10
Sleep disorder	327, 780.5	G47
Seizure/Epilepsy	345	G40
Traumatic brain injury (TBI)/Concussion	800, 801, 803, 804, 850, 851, 852, 853, 854	S02.0, S02.1, S02.8, S02.9, S08.0, S06, S07
Asthma	493	J45

© 2026 Fontanella CA et al. *JAMA Network Open*.

eTable 4. Deliberate Self-Harm ICD Codes.

ICD-9	ICD-10
E950-E959	T1491, T43012, T43022, T431X2, T43202, T43212, T43222, T43292, T433X2, T434X2, T43502, T43592, T43602, T424X2, T43622, T43632, T438X2, T4392, T360X2, T361X2,T362X2, T363X2,T364X2, T365X2, T366X2, T367X2,T368X2, T370X2,T371X2, T372X2, T373X2, T374X2, T375X2, T378X2,T380X2, T381X2, T382X2, T383X2, T384X2, T385X2, T386X2, T387X2, T391X2, T392X2, T394X2, T398X2,T403X2, T410X2, T411X2, T413X2, T415X2, T420X2, T421X2, T422X2, T423X2, T425X2, T426X2, T428X2,T440X2, T441X2, T442X2, T443X2, T444X2, T445X2, T446X2, T447X2, T448X2, T450X2, T451X2, T452X2, T453X2, T454X2, T457X2, T458X2, T460X2, T461X2, T462X2, T463X2, T464X2, T465X2, T466X2, T467X2, T468X2, T470X2, T471X2, T472X2, T473X2, T474X2, T475X2, T476X2, T477X2, T478X2, T480X2, T481X2, T483X2, T484X2, T485X2, T486X2, T490X2, T491X2, T492X2, T493X2, T494X2, T495X2, T496X2, T497X2, T498X2, T500X2, T501X2, T502X2, T503X2, T504X2, T505X2, T506X2, T507X2, T508X2, T510X2, T511X2, T512X2, T513X2, T520X2, T521X2, T522X2, T523X2, T524X2, T528X2, T530X2, T531X2, T532X2, T533X2, T534X2, T535X2, T536X2, T537X2, T535X2, T536X2, T537X2, T540X2, T541X2, T542X2, T543X2, T550X2, T551X2, T560X2, T561X2, T562X2, T563X2, T564X2, T565X2, T566X2, T567X2, T570X2, T571X2, T572X2, T573X2, T578X2, T582X2, T588X2, T590X2, T591X2, T592X2, T593X2, T594X2, T595X2, T596X2, T597X2, T600X2, T601X2, T602X2, T603X2, T604X2, T608X2, T618X2, T620X2, T621X2, T622X2, T628X2, T632X2, T650X2, T651X2, T653X2, T654X2, T655X2, T656X2, T3692, T3792, T3992, T400X2, T402X2, T401X2, T405X2, T40602, T40692, T404X2, T408X2, T40902, T40992, T4142, T4272, T4392, T4592, T4792, T4992, T5292, T5392, T5492, T5692, T5792, T5802, T5812, T5892, T5992, T6092, T6102, T6112, T6192, T6292, T6392, T6402, T6482, T6592, T38802, T38812,<br>T38892, T38902, T38992, T39012, T39092, T39312, T39392, T407X2, T40712, T41202, T41292, T43602, T43612, T43642, T43692, T44902, T44992, T45512, T45522, T45602, T45612, T45622, T45692, T46902, T46992, T48202, T48292, T48902, T48992, T50902, T50992, T50A12, T50A22, T50A92, T50B12, T50B92, T50Z12, T50Z92, T510X2, T510X2, T518X2, T5192, T56812, T56892, T59812, T59892, T61772, T61782, T63002, T63012, T63022, T63032, T63042, T63062, T63072, T63082, T63092, T63112, T63122, T63192, T63302, T63312, T63322, T63332, T63392, T63412, T63422, T63432, T63442, T63452, T63462, T63482, T63512, T63592, T63612, T63622, T63632, T63692, T63712, T63792, T63812, T63822, T63832, T63892, T65212, T65222, T65292, T65812, T65822, T65832, T65892T58 or T59 and 6TH character of ‘2’, T71112, T71122, T71132, T71152, T71162, T71192, T71222, T71232, X71-83

© 2026 Fontanella CA et al. *JAMA Network Open*.

eTable 5. Other Individual and Family Variables and ICD Codes.

Variable	ICD-9	ICD-10
Educational/Occupational Problem	V62.0: Unemployment<br>V62.1: Adverse effects of work environment<br>V62.2: Other occupational circumstances or maladjustment<br>V62.3: Educational circumstances	Z55: Problems related to education/literacy<br>Z56.0-Z56.6: General problems related to employment and unemployment<br>Z56.89: Other problems related to employment<br>Z56.9: Unspecified problems related to employment
History of Abuse/Neglect	995.5: Child maltreatment syndrome<br>V15.41: History of physical abuse<br>V15.42: History of emotional abuse<br>V15.49: Other psychological trauma<br>V61.11: Counseling for victim of spousal and partner abuse<br>V61.21: Counseling for victim of child abuse	T74: Adult and child abuse, neglect, and other maltreatment, confirmed<br>T76: Adult and child abuse, neglect, and other maltreatment, suspected<br>Z04.4: Encounter for examination and observation following alleged rape<br>Z04.7: Encounter for encounter for examination and observation following alleged physical abuse<br>Z61.4: Problems related to alleged sexual abuse of child by person within primary support group<br>Z61.5: Problems related to alleged sexual abuse of child by person outside primary support group<br>Z61.6: Problems related to alleged physical abuse of child<br>Z62.810: Personal history of physical and sexual abuse in childhood<br>Z62.811: Personal history of psychological abuse in childhood<br>Z62.812: Personal history of neglect in childhood<br>Z62.813: Personal history of forced labor or sexual exploitation in childhood<br>Z62.819: Personal history of unspecified abuse in childhood<br>Z69.010: Encounter for mental health services for victim of parental child abuse<br>Z69.020: Encounter for mental health services for victim of non-parental child abuse<br>Z69.11: Encounter for mental health services for victim of spousal or partner abuse<br>Z69.81: Encounter for mental health services for victim of other abuse

© 2026 Fontanella CA et al. *JAMA Network Open*.

Family Relational Problem	V61.01: Family disruption due to family member on military deployment<br>V61.02: Family disruption due to return of family member from military deployment<br>V61.03: Family disruption due to divorce or legal separation<br>V61.04: Family disruption due to parent-child estrangement<br>V61.08: Family disruption due to other extended absence of family member<br>V61.09: Other family disruption<br>V61.23: Counseling for parent-biological child problem<br>V61.24: Counseling for parent-adopted child problem<br>V61.29: Other parent-child problems<br>V61.3: Problems with aged parents or in-laws<br>V61.49: Other health problems within the family<br>V61.8: Other specified family circumstances<br>V61.9: Unspecified family circumstances	Z62.820: Parent-biological child conflict<br>Z62.821: Parent-adopted child conflict<br>Z62.832: Non-relative guardian-child conflict<br>Z62.890: Parent-child estrangement NEC<br>Z62.891: Sibling rivalry<br>Z62.898: Other specified problems related to upbringing<br>Z62.9: Problem related to upbringing, unspecified<br>Z63.0: Problems in relationship with spouse or partner<br>Z63.1: Problems in relationship with in-laws<br>Z63.31: Absence of family member due to military deployment<br>Z63.32: Other absence of family member<br>Z63.5: Disruption of family by separation and divorce<br>Z63.71: Stress on family due to return of family member from military deployment<br>Z63.79: Other stressful life events affecting family and household<br>Z63.8: Other specified problems relating to primary support group<br>Z63.9: Problem related to primary support group, unspecified
Economic and Housing Problem	V60.0: Lack of housing<br>V60.1: Inadequate housing<br>V60.2: Inadequate material resources<br>V60.89: Other specified housing or economic circumstances<br>V60.9: Unspecified housing or economic circumstances	Z59.0: Homelessness<br>Z59.1: Inadequate housing<br>Z59.4: Lack of adequate food and drinking water<br>Z59.5: Extreme poverty<br>Z59.6: Low income<br>Z59.7: Insufficient social insurance and welfare support<br>Z59.8: Other problems related to housing and economic circumstances<br>Z59.9: Problem related to housing and economic circumstances, unspecified
Exposure to Violence (excluding abuse)	E96.0: Fight brawl rape<br>E96.1: Assault by corrosive or caustic substance, except poisoning<br>E96.2: Assault by poisoning	X92: Assault by drowning and submersion<br>X93: Assault by handgun discharge<br>X94: Assault by rifle, shotgun and larger firearm discharge

© 2026 Fontanella CA et al. *JAMA Network Open*.

	E96.3: Assault by hanging and strangulation<br>E96.4: Assault by submersion<br>E96.5: Assault by firearms and explosives<br>E96.6: Assault by cutting and piercing instrument<br>E96.8: Assault by other and unspecified means<br>E96.9: Late effects of injury purposely inflicted by other person<br>E97.0: Injury due to legal intervention by firearms<br>E97.1: Injury due to legal intervention by explosives<br>E97.2: Injury due to legal intervention by gas<br>E97.3: Injury due to legal intervention by blunt object<br>E97.4: Injury due to legal intervention by cutting and piercing instrument<br>E97.5: Injury due to legal intervention by other unspecified means<br>E97.6: Injury due to legal intervention by unspecified means<br>E97.7: Late effects of injuries due to legal intervention	X95: Assault by other and unspecified firearm and gun discharge<br>X96: Assault by explosive material<br>X97 Assault by smoke, fire and flames<br>X98: Assault by steam, hot vapors and hot objects<br>X99: Assault by sharp object<br>Y00: Assault by blunt object<br>Y01: Assault by pushing from high place<br>Y02: Assault by pushing or placing victim in front of moving object<br>Y03: Assault by crashing of motor vehicle<br>Y04: Assault by bodily force<br>Y08: Assault by other specified means<br>Y09: Assault by unspecified means<br>Y35: Legal intervention injury

© 2026 Fontanella CA et al. *JAMA Network Open*.

eTable 6. Contextual Level Variable Sources and Yearly Availability.

Variable Name	Data Source	Available Years	Yearly Modifications
Rural-Urban Continuum Codes	Economic Research Service, US Department of Agriculture	2013	2013 data were used for all years.
Religious Establishments	County Business Patterns: Religious Organization (NAICS = 813110)	2012-2019	2012-2019 data were used to estimate 2010-2011 by regression.
Crime Rates	Federal Bureau of Investigation Uniform Crime Reporting Program Data	2010-2014, 2016-2019	2010-2014, 2016-2019 data were used to estimate 2015 data by regression.
Population[a]	National Cancer Institute Surveillance, Epidemiology, and End Results Program, U.S. County Population Data	2010-2019	
Social Vulnerability Index	Centers for Disease Control and Prevention, Agency for Toxic Substances and Disease Registry	2014, 2016, 2018, 2020	2014 data was used for 2010-2014; 2016 data was used for 2015-2016; 2018 data was used for 2017-2019

[a]Population data for all ages was used to calculate religious establishments and crime rates per 100,000 people.

© 2026 Fontanella CA et al. *JAMA Network Open*.

eTable 7. Missingness of Contextual Level Variables.

Variable Name	Missing count	Missing percentage
RUCC	17	0.2%
Social vulnerability index	NA[a]	NA[a]
Religious establishments	22	0.2%
Crime rates	918	9.5%

[a] Counts and percentages not shown due to small cell count < 11.

© 2026 Fontanella CA et al. *JAMA Network Open*.
