# Supplementary material for: Suicide Risk and Protective Factors Among Medicaid-Enrolled Black Youth With a Mental Health Diagnosis
Source: JAMA Netw Open. 2026 Feb 18;9(2):e2559657. doi: 10.1001/jamanetworkopen.2025.59657 (PMC12917685; doi:10.1001/jamanetworkopen.2025.59657)
Supplement: Supplement 2. — Data Sharing Statement [file jamanetwopen-e2559657-s002.pdf]

Data Sharing Statement

Fontanella. Risk and Protective Factors and Suicide Among Medicaid-Enrolled Black Youth With a Mental Health Diagnosis. *JAMA Netw Open*. Published February 18, 2026. doi:10.1001/jamanetworkopen.2025.59657

Data
Data available: No

Additional Information
Explanation for why data not available: Data are protected under a DUA and unable to be shared.
